# Smooth Faced Pluggers

**Published:** 1872-11

**Authors:** G. O. Howard


### ARTICLE III.

### *Smooth Faced Pluggers.*

BY DR. G. O. HOWARD.

Read before the Illinois State Dental Society.

Persons who have never written upon a new subject, or at least new to them, can hardly imagine the difficulty to be encountered. It is easy to write upon almost any subject that has been discussed, because ideas that are suggested will lead to others that are different, and perhaps new; but with a new subject, one that has not been handled, we must depend entirely upon ourselves; we cannot get ideas from others to expand, nor refer to others for proof. I have selected for this paper two subjects, which, one year ago, were entirely new, (to me, at least.) I thought when I promised our executive committee that I would furnish a paper upon these subjects, that I should have no difficulty in doing so; since that time I have changed my mind and practice with these subjects, and I have concluded that it is with them as with everything else, viz: the more I know the less I think I know.

I was led to the use of smooth-faced pluggers a little less than a year ago by observing the imperfections in my fillings; they were pock-marked, little particles of gold were broken out, especially upon that part of the filling used most in mastication. I would finish them down again only to find them in the same condition in a very short time; my first impression was that it was caused by the gold I used, or the manner in which I used it. With that idea I prepared the gold in all the ways that I could think of. I folded it into ribbons, rolled it into ropes, made blocks and pledgets, used heavy foils, also corrugated gold; but all to no improvement. My fillings would all, in time, catch the small-pox. I then concluded that it must be the fault of my instruments. In arriving at this, I had first necessarily to understand the natural laws that govern the welding together of the two pieces of gold. I found that it was done by the aid of the two laws of adhesion and cohesion; of these cohesion is of the greater importance to us, where we are using gold foil; adhesion assists in our operation by kindly sticking one piece of gold to another long enough for us to pick up our plugger and weld them together, and that is all. A perfect welding of one piece of gold to another is our object, and this is done the same with gold as with any other metal that can be welded—iron for instance. The blacksmith first prepares the pieces to be welded by hammering them out smooth, then adding some borax to clean the parts to be welded; then heats as much as necessary, and with his hammer welds them into one piece; they are a coherent body. Cohere is to cling together, as the fibres of one substance when force is applied to break it.

Ever since the dental profession abandoned the method of packing gold by wedging one piece so as to hold another, they have depended upon cohesion for their success, although we hear a good deal about adhesion; but that is a secondary law in packing gold. Now if we pack gold under the law of cohesion, (and I am thoroughly convinced by experiments that we do,) then the more coherent our filling (that is the more solid) the better it is. While I was thinking of this matter, I was doing some work for a blacksmith; a man of intelligence and a good workman. I asked him why blacksmiths did not use a hammer with serrated face to weld iron with, the same as our instruments were that we used to weld gold. He thought a moment, and said, "if we were to use serrated faced hammers our welding would be full of holes." Now, if this be true with iron, it must be true with gold. Acting under that idea, I picked up my burnisher, (the one that came in Atkinson's set,) and run over the filling that I was then at work upon, and made it level and smooth burnished, then annealed a piece of foil, laid it upon the filling, and with the burnisher packed it. Upon examination I found to my surprise and delight that it could not be removed—it was a part of the filling—and by magnifying I found it to be perfect; it was level, smooth and even burnished. I immediately took the pluggers that I generally used and removed the serration from their points, and have used them ever since with the most gratifying results. From that day to this my fillings have been free from that almost sickening appearance of being pock marked. If any person doubts that the smooth pointed plugger is superior to the serrated I will be pleased to have them follow me through the following:

Suppose we were filling a large cavity upon the grinding surface of a molar tooth, and are using serrated points; we will work away and pack the gold as thoroughly as possible, sparing no amount of force to make the gold solid at every point until the cavity is half full; we will then stop and examine it thoroughly; we find a body of gold that is just as hard as we can make it; it presents a surface of hills and valleys; the hills are hard and so are the valleys. We pick up the next piece of gold and lay it to the filling, and if we could place the serrates of our plugger on it, so that they would correspond with the hills and valleys in the surface of the filling, we would be making a perfect coherent body of gold; but instead of that we find it impossible to do so with any certainty; and when we do happen to do it we do not know it, and the probabilities are that ninety-nine times out of every hundred our instrument goes down in the wrong place; the result is that we head over more or less of those small valleys; this may not do any harm, but I am inclined to believe that it does; we will go on until the cavity is filled, then trim and finish. Now, is there a dentist here that believes that to be a perfect filling? Do you suppose that the sides of that filling next to the walls of the cavity are perfect? If you do let me assure you that you are mistaken, for it is impossible to make it so with a serrated point. In a short time we find the surface all full of little holes, and we have the dissatisfaction of knowing that our work does not stand as we left it.

Now, suppose on the other hand, we were filling a similar cavity with smooth points. We will work just as carefully as with the other until we reach about the same point in the filling. Upon examination we find a body of gold that is perfectly level and smooth, almost burnished. In laying on the next piece of gold and driving it home with the smooth-faced plugger, we bring the two parts of gold perfectly together without any difficulty; there is no danger of heading over any little valleys, as there was in the other case, because the valleys are not there. We finish the fillings, and now we have a perfectly solid piece of gold from foundation to top, as much so as if we had cast it into the cavity while in a molten state; with the serrated points we had a honey-combed body of gold, which was not worth a five cent piece only to remove and sell for old gold. Some may say, if we were using fine serrated points like "Varney's" for instance, we would not find the difficulty. Fine serrated points are better than coarse, I will admit, but the principle is the same in the one as the other. Others will say that their pluggers are worn down smooth, or nearly so. The danger is fully as great, if not greater. in using that class of instruments, for in packing gold with them, we cannot keep the surface level, because their points being worn, are oval; and we meet with the same difficulty as with serrated points, only with the latter the hills and valleys are equal and of the same size; with the former the valleys are larger than the hills; hence the more danger. We must have the points flat and smooth, with sharp edges; then our filling will be level and smooth, with ordinary care. With the sharp edge smooth point, gold is handled as easily, after a little practice, as with serrated points; for with either we use the edge and not the face in handling gold.

A few words in regard to the economy in using smooth points, and then I am done with this part of my paper. First, the saving of time: I find when I am through packing my gold that it does not take near the time to finish that it did when I used the serrated points. Second, it does not cost me as much for pluggers as it used to. I am now using a set of Atkinson's pluggers, which a year ago I was thinking I must send and get re serrated; but since using the smooth point, all I have to do when they are a little dull, is to take out my oil stone and sharpen them, clean off the oil, and go ahead.

